# Using Manipulated Photographs to Identify Features of Streetscapes That May Encourage Older Adults to Walk for Transport

**DOI:** 10.1371/journal.pone.0112107

**Published:** 2014-11-14

**Authors:** Jelle Van Cauwenberg, Veerle Van Holle, Ilse De Bourdeaudhuij, Peter Clarys, Jack Nasar, Jo Salmon, Liesbet Goubert, Benedicte Deforche

**Affiliations:** 1 Department of Human Biometry and Biomechanics, Vrije Universiteit Brussel, Brussels, Belgium; 2 Department of Movement and Sport Sciences, Ghent University, Ghent, Belgium; 3 Fund for Scientific Research Flanders (FWO), Brussels, Belgium; 4 City and Regional Planning, The Ohio State University, Columbus, Ohio, United States of America; 5 School of Exercise and Nutrition Sciences, Deakin University, Melbourne, Victoria, Australia; 6 Department of Experimental–Clinical and Health Psychology, Ghent University, Ghent, Belgium; Örebro University, Sweden

## Abstract

Experimental evidence of environmental features important for physical activity is challenging to procure in real world settings. The current study aimed to investigate the causal effects of environmental modifications on a photographed street's appeal for older adults' walking for transport. Secondly, we examined whether these effects differed according to gender, functional limitations, and current level of walking for transport. Thirdly, we examined whether different environmental modifications interacted with each other. Qualitative responses were also reported to gain deeper insight into the observed quantitative relationships. Two sets of 16 panoramic photographs of a streetscape were created, in which six environmental factors were manipulated (sidewalk evenness, traffic level, general upkeep, vegetation, separation from traffic, and benches). Sixty older adults sorted these photographs on appeal for walking for transport on a 7-point scale and reported qualitative information on the reasons for their rankings. Sidewalk evenness appeared to have the strongest influence on a street's appeal for transport-related walking. The effect of sidewalk evenness was even stronger when the street's overall upkeep was good and when traffic was absent. Absence of traffic, presence of vegetation, and separation from traffic also increased a street's appeal for walking for transport. There were no moderating effects by gender or functional limitations. The presence of benches increased the streetscape's appeal among participants who already walked for transport at least an hour/week. The protocols and methods used in the current study carry the potential to further our understanding of environment-PA relationships. Our findings indicated sidewalk evenness as the most important environmental factor influencing a street's appeal for walking for transport among older adults. However, future research in larger samples and in real-life settings is needed to confirm current findings.

## Introduction

The benefits of promoting physical activity (PA) to foster physical, mental, and social health in the growing, but underactive, Western older populations (≥65 years) are well established [1], [2]. Regular walking for transport (to do grocery shopping or visit a friend) is particularly relevant in this age group as it is healthy, enjoyable, cheap, accessible, environmentally friendly, and easy to integrate into older adults' daily routines [3]–[5]. To effectively promote walking for transport among older adults, supportive built or physical environments should be available and accessible [6]. The presence of a supportive environment is considered especially relevant for older compared to younger-aged adults, since age-related functional limitations might increase difficulties in overcoming environmental barriers [7]–[9]. In several studies, access to a variety of daily destinations (such as grocery stores, bank offices, parks and libraries) appeared to be a consistent correlate of older adults' walking for transport [10]–[13]. For example, Frank and colleagues [13] reported US older adults living in neighborhoods with easy access to destinations (so-called ‘high-walkable’ neighborhoods) to be twice as likely to walk for transport compared to residents of neighborhoods with difficult access to destinations (‘low-walkable’ neighborhoods). Despite its potential to promote older adults' walking for transport, access to destinations is a macro-scale environmental factor influenced by multiple economic actors and local, regional and central authorities and is, therefore, difficult to change in existing neighborhoods [14].

Qualitative studies highlight the importance of micro-scale environmental factors, such as sidewalk evenness, presence of vegetation, and upkeep [15]–[18]. These micro-scale factors are mostly under influence of local actors and, therefore, more amenable to change [14]. This makes micro-scale environmental factors promising intervention targets aimed at increasing walking for transport among older adults. However, quantitative observational studies on the relationships between micro-scale environmental factors and older adults' walking for transport have had inconsistent findings [19], [20]. These inconsistencies are potentially due to a lack of heterogeneity in the environments being studied [21]; inaccurate definitions of a ‘local neighborhood’ for older adults [22], [23]; and the use of questionnaires that require older adults to recall their environmental perceptions and experiences while not in that environment [24]. Furthermore, environmental co-variation, the tendency of environmental factors to co-occur, makes it difficult to differentiate the influence of each individual environmental factor [25]. An additional limitation of evidence to date is the observational nature of the studies; identifying a correlation between certain features of the environment and PA does not provide insight into potential causal associations. Experimental approaches are needed to better understand these relationships [19], [26], [27].

The use of photographs and specialized software (such as Adobe Photoshop) enables relatively easy manipulation of environmental factors in photographed street environments and controlled investigation of their effects on the streets' appeal for walking for transport. This approach has been proposed previously [28] and used in research on PA among children in playgrounds [29] and neighborhood preference among adults [30], but has not been applied to study relationships between physical environments and older adults' walking for transport. The use of manipulated photographs in an experimental setting allows the researcher to carefully create photographs of streetscapes that capture the whole range of variation in the manipulated environmental factors and control for environmental co-variation between these factors. Furthermore, the need to define an older adults' ‘local neighborhood’ and reliance on recall is eliminated as exposure to and assessment of the environment occurs simultaneously and consistently between participants.

To tailor environmental interventions to the needs of specific subgroups of the older population, knowledge of individual factors (e.g. gender, functional limitations, and current walking for transport level) that may moderate environment-PA relationships is required [31]. For example, press-competence models hypothesize that environment-PA relationships are stronger among older adults with higher levels of functional limitation compared with those with lower levels [32]. Another potentially relevant moderator is current walking for transport level [31]. Identifying whether there are unique environmental factors that appeal to non- or low-walkers (compared to regular walkers) is important for informing the design of streetscapes that may encourage the least active to walk more. Knowledge regarding the moderators of associations between micro-scale environmental features and walking for transport is limited [19].

As already noted, the relationship between particular environmental factors and older adults' walking for transport might interact with other environmental factors. For example, the presence of greenery might not matter in an older adults' decision to walk for transport when the available sidewalk is of poor quality. However, in a street with a high quality sidewalk the presence of greenery might further increase the street's appeal for walking. These interactions between environmental factors are described in Alfonzo's “Hierarchy of Walking Needs” [33]. In this model, environmental factors are categorized into four hierarchical walking needs: (1) accessibility (e.g. distance to destinations, presence of a sidewalk); (2) comfort (e.g. sidewalk evenness, separation from traffic, benches); (3) safety from crime (e.g. surveillance, hiding places); and (4) pleasurability (e.g. vegetation, historic elements, mixed land use) [33]. Alfonzo hypothesized that lower order needs (e.g. pleasurability) do not affect walking for transport as long as the higher order needs (i.e. accessibility, comfort, and safety from crime) are not fulfilled. To our knowledge, no previous research has investigated interaction effects between environmental factors with respect to older adults' walking for transport.

The current study aimed to investigate the causal effects of environmental modifications on a photographed street's appeal for older adults' walking for transport. Secondly, we examined whether these effects differed according to gender, functional limitations, and current level of walking for transport. Thirdly, we examined whether different environmental modifications interacted with each other. Qualitative responses were also reported to gain deeper insight into the observed quantitative relationships.

## Materials and Methods

The present study used quantitative and qualitative methods to determine which, and also how and why, environmental factors influence a street's appeal for walking for transport.

### Participants

Purposeful convenience sampling was used to recruit 60 Flemish older adults. We aimed to include 50% women and physically active as well as inactive older adults. Older family members and relatives of the research team were contacted and invited to participate. Snowball sampling was used to recruit additional participants. For inclusion in the study, the participants had to be: 65 years or older and retired, community dwelling, able to walk independently, and reside in an urban (>600 inh./km^2^) or semi-urban (300–600 inh./km^2^) municipality [34].

### Protocol

After initial contact and agreement to participate, a trained researcher visited the participant at home. The researcher explained the protocol and obtained written informed consent (including permission to use de-identified quotes in research publications). The home visit took approximately 45 minutes and consisted of two parts: a structured interview and two sorting tasks each including 16 manipulated panoramic photographs. Data collection was performed in March-April 2013. The study protocol was approved by the ethical committees of the Brussels University hospital and Ghent University hospital.

### Structured interview

The structured interview collected demographic information (i.e., age, gender, place of birth, marital status, car ownership, educational level, and former main occupation), functional limitations, and physical activity. The physical functioning scale of the validated Short-Form 36-item Health Survey was used to assess functional limitations [35], [36]. Participants were asked to indicate how their health limited their ability to perform ten activities of daily living (e.g. climbing stairs, washing and dressing, etc.) on a 3-point scale: severely, somewhat, or not limited. Activities in which participants reported being severely or somewhat limited were summed to create the variable ‘number of functional limitations’. This variable was dichotomized based on the median into: no or one functional limitation (coded 0) and two or more functional limitations (coded 1).

To assess engagement in different PA domains and total PA, the International Physical Activity Questionnaire (IPAQ, long form, last 7 days, interview version) was used. The IPAQ has been validated in older adults [37] and has been used in several previous studies in older adults [12], [38], [39]. Standard scoring procedures (available on http://www.ipaq.ki.se/) were followed to calculate weekly minutes of walking for transport and moderate-to-vigorous physical activity (MVPA). Weekly minutes of walking for transport was dichotomized into: 0–60 minutes of walking for transport (coded 0), and >60 minutes of walking for transport (coded 1). We labelled this variable ‘current walking for transport level’. Participants were classified as meeting the PA recommendations if they reported a minimum of 150 minutes MVPA/week [1].

### Development of manipulated photographs

**Figure 1:**
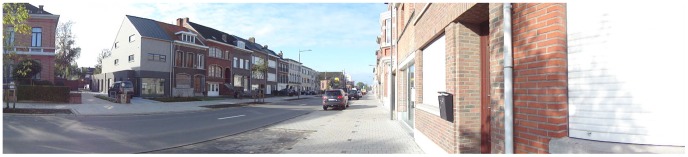
The basic panoramic photograph that served as a basis for all environmental manipulations.

The panoramic photographs were all modified versions of one “basic” panoramic photograph (see Figure 1). This basic photograph was taken at eye level from the sidewalk in a typical (semi-)urban street in Flanders (Belgium). The basic photograph itself was not included in the two sets of photographs, because it was necessary to modify it slightly to be able to perform the intended manipulations. In both sets of 16 photographs, four environmental factors were experimentally manipulated using Adobe Photoshop software. Each environmental factor had two levels, yielding 2^4^ = 16 photographs per sorting task that presented all possible combinations of environmental factors. We restricted the number of photographs to 16 per set since 16 photographs appeared to be the maximum number of photographs that was feasible for older participants to sort during a pilot test of our protocol. The selection of environmental factors to be manipulated was based upon the environmental factors that appeared to be key factors affecting older adults' walking for transport in two previous studies with Flemish older adults [15], [40]. The two sets of sixteen manipulated photographs were printed in color on cardboard in a 27.0×7.7 cm format.

**Figure 2:**
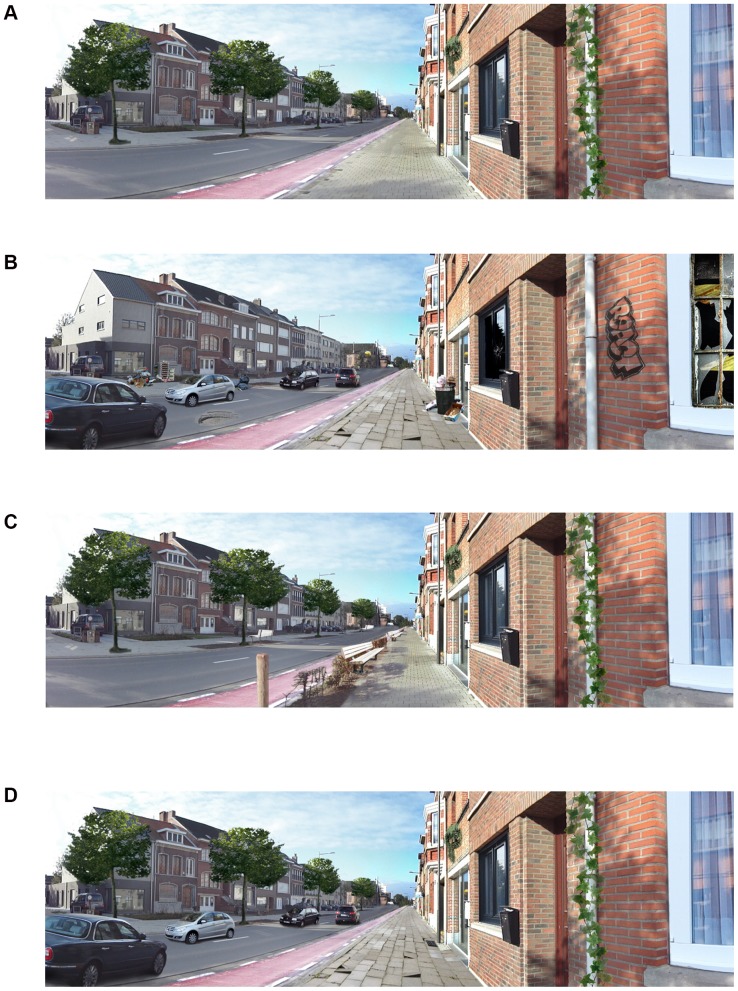
The anticipated best and worst streets for walking for transport in sorting task A and B. Anticipated best and worst streets of the 16 streets in each sorting task. A: The anticipated best street for walking for transport in sorting task A with an even sidewalk, no traffic, good upkeep, and vegetation. B: The anticipated worst street for walking for transport in sorting task A with an uneven sidewalk, traffic, bad upkeep, and no vegetation. C: The anticipated best street for walking for transport in sorting task B with an even sidewalk, no traffic, benches present, and sidewalk separated from traffic by a hedge. B: The anticipated worst street for walking for transport in sorting task B with an uneven sidewalk, traffic present, no benches, and no separation from traffic.

In the first sorting task (sorting task A), the four manipulated environmental factors potentially important for older adults' walking for transport included: sidewalk evenness (0 =  uneven, 1 =  even), traffic (0 =  traffic present, 1 =  no traffic), overall upkeep (0 =  bad upkeep, 1 =  good upkeep), and vegetation (0 =  no vegetation, 1 =  vegetation present). For the environmental factor “overall upkeep”, the level “bad upkeep” included the presence of garbage, broken windows, graffiti, and a pothole in the street surface. Figure 2A presents the manipulated photograph containing all factors anticipated to be most favorable for walking for transport (even sidewalk, no traffic, good upkeep, and vegetation present). Figure 2B presents the least favorable street for walking for transport (uneven sidewalks, traffic present, bad upkeep, and no vegetation). The 14 remaining photographs depicted all other possible combinations of these four environmental factors.

Sorting task B included four environmental factors related to walking facilities and traffic safety: sidewalk evenness (0 =  uneven, 1 =  even), traffic (0 =  traffic present, 1 =  no traffic), benches (0 =  no benches, 1 =  benches present), and separation from traffic (0 =  no separation, 1 =  sidewalk separated from traffic by a hedge). Figure 2C presents the manipulated photograph containing all favorable factors for walking for transport (even sidewalk, no traffic, benches present, and sidewalk separated from traffic by a hedge). Figure 2D presents the anticipated worst street for walking for transport (uneven sidewalk, traffic present, no benches, and no separation).

### Sorting task with manipulated photographs

During the second part of the home visit, each of the participants performed two sorting tasks with the 16 manipulated photographs [see additional file 1]. Responses to color photographs have been shown to accurately reflect on-site responses to real environments [28], [41]. To control for order effects, each participant alternately began with sorting task A or B. First, the printed photographs were shuffled and placed on a table in front of the participant in random order. The researcher read the following instructions: “Imagine yourself walking to a friend's home located 10 minutes from your home during daytime. The weather is ideal for walking, it is not too warm, not too cold, there is no wind, and it is not raining. You are feeling well today and you have no unusual physical problems that hinder your walking. You see sixteen photographs. Each photograph depicts the same street, but you will notice that certain things differ from photograph to photograph. Please take your time to look at the photographs, we need you to pick out the street or streets that invite you least and most to walk to your friend's home. So, you choose the worst and best street, and if you think that several streets are equally bad or good, you can pick several photographs as worst or best. We ask you to think aloud when you choose the photographs so that we know why you selected certain photographs. There is no good or bad solution, we are just interested in what you consider as most important while walking to your friend's home.”

By telling the participants to imagine themselves walking to friend's home located ten minutes from their own home, a specific context was provided [42] and accessibility (i.e. distance to the destination) was standardized. When the participants had chosen the worst and best street(s), the researcher put these photographs aside and collected the remaining photographs. Next, the researcher placed seven cards on the table. From left to right, these cards depicted a score ranging from zero (least appealing) to six (most appealing). The street(s) that was/were chosen as the least appealing to walk along was/were placed underneath score zero (least appealing). The street(s) that was/were chosen as the most inviting to walk along was/were placed underneath score six (most appealing).

Then, the researcher read the following instructions: “I have placed the photograph(s) that you picked as least appealing underneath score zero, your most appealing street(s) underneath score six. Now, we need you to sort the remaining photographs from least to most appealing by allocating them a score from zero to six, with zero as the least and six as the most appealing street to walk to your friend's home. You can place several streets underneath the same score and you can swap the order of the photographs at any time. The streets that already received a score of zero or six can also be changed and you can add other photographs to these scores. We ask you to think aloud when you sort the photographs so that we know why you sort the photographs in that manner. Again, there is no good or bad solution, we are just interested in what you consider as most important while walking to your friend's home.” When the participant had completed the sorting task, the researcher asked him or her to check the sorting task and to make sure that the streets' score increased from zero to six and that equally appealing streets received the same score.

Lastly, the researchers collected qualitative information using a voice recorder on the perceived influence of the manipulated environmental factors on the appeal of the street for walking for transport. Participants were asked to describe the reasons why they sorted the photographs as they did. Follow-up questions were asked (e.g., ‘Which of these two factors is most important for you?’, ‘Why do you consider this factor more important?’) to obtain more details about the relative importance of the various environmental factors for transport-related walking. The same protocol was then repeated for the other set of 16 photographs.

### Analyses

#### Quantitative analyses

From each sorting task, there were 960 scores (16 streets * 60 participants) ranging from zero to six, which acted as the dependent variables (see Dataset S1). These 960 scores were not independent from each other, responses from the same participant and responses to the same street can be anticipated to be correlated. To adjust for this clustering of scores within participants and streets, multilevel cross-classified linear regression models were applied using MLwiN 2.28 [43]. All analyses were performed separately for the two sorting tasks. Model parameter estimates were obtained via Markov Chain Monte Carlo (MCMC) procedures applying an orthogonal parameterization [44]. The analyses consisted of three consecutive steps. Firstly, the univariate relationships between each separate individual and environmental factor and the awarded scores were analyzed. Secondly, a basic model was constructed including all four environmental factors simultaneously, allowing the independent adjusted effects of each of the four environmental factors to be examined. This basic model also included the main effects of the individual factors that yielded significant effects in the first step. The interaction (moderating) effects of gender, functional limitations, and current walking for transport level on associations between the environmental factors and the awarded scores were then added separately to the basic model. Similarly, the interaction effects between the environmental factors were examined. All significant interaction effects observed in the second step were then entered simultaneously into the basic model. A final model was constructed by allowing random slopes that improved the model fit and by deleting non-significant effects that did not improve the model fit. Models were compared using the Deviance Information Criterion [44]. The significance level was determined at alpha  = .05.

#### Qualitative analyses

The qualitative information was used to add depth and understanding to the quantitative relationships. The recorded qualitative information was transcribed verbatim. For the analysis of the qualitative data, the framework approach as described by Pope et al. [45] was followed. The transcripts were read thoroughly to become familiarized with the data. Then, since our experiment had six manipulated environmental factors, the reasons for sorting the photographs were categorized into these six *a priori* themes. Since sidewalk evenness and presence of traffic were manipulated in both sorting tasks, qualitative data from the two tasks were combined for these two factors. Nvivo 9 Software (QSR International) was used to facilitate the categorization. Finally, all information per theme was summarized and participants' quotes were used to illustrate our findings.

## Results

### Sample characteristics

Participants had a mean age of 74.1±6.2 years and 48.3% were women. The majority of participants were born in Belgium (96.7%), married (73.3%), and owned at least one car (83.3%). Approximately one-third of participants (31.7%) held a tertiary education degree and 55.1% held a white collar job. Approximately half the participants (51.7%) reported being functionally limited in two or more activities of daily living, 46.7% met the PA recommendations, and 25.0% reported to have walked for transport for more than 60 minutes in the past seven days.

### Quantitative results for sorting task A

For sorting task A, all environmental factors were significantly and positively related to the street's appeal score (see Table 1). The largest effect was observed for sidewalk evenness; streets with an even sidewalk were assigned 2.53 points more (on a maximum of 6) compared to streets with an uneven sidewalk. Streets without traffic were awarded 0.57 points more compared to streets with traffic. Similarly, streets with good upkeep received 0.92 points more compared to streets with bad upkeep. A significant interaction effect between sidewalk evenness and overall upkeep was found: the positive effect of good upkeep was significantly stronger in a street with an even compared to an uneven sidewalk. When sidewalks were uneven, good overall upkeep resulted in an increase of 0.92 points. However, when sidewalks were even, good overall upkeep resulted in an increase of 1.77 points. A significant positive main effect was also observed for the presence of vegetation. No significant interaction effects between environmental and individual factors were observed.

**Table 1:** Results for the main and interaction effects of the environmental factors with individual and other environmental factors for sorting task A^1^.

	B	S.E.	P
Intercept	0.14	0.14	
**Main effects individual factors**			
Age (G.M.)	0.02	0.01	0.04
**Main effects environmental factors** 2			
Sidewalk evenness	2.53	0.19	<0.001
Traffic	0.57	0.10	<0.001
Overall upkeep	0.92	0.18	<0.001
Vegetation	0.56	0.13	<0.001
**Interaction effects between environmental factors**			
Sidewalk evenness*upkeep	0.85	0.20	<0.001

S.E. =  standard error; G.M. =  centered around its grand mean.

1 In sorting task A one streetscape was manipulated on four environmental factors; sidewalk evenness, traffic, overall upkeep and vegetation. Sixty older adults sorted the streetscapes on their appeal for walking for transport using a seven-point scale. The B's can be interpreted as the effect of the environmental modification on this seven-point scale.

2 The reference categories for the environmental factors were the anticipated negative aspects of the factor (i.e. uneven sidewalk, bad upkeep, no vegetation, and traffic present).

### Quantitative results for sorting task B

All environmental factors yielded significant and positive main effects on the scores, with the exception of the presence of benches (see Table 2). Streets with an even sidewalk received on average 3.23 points more compared to streets with an uneven sidewalk. Streets without any traffic also received significantly higher scores compared to streets in which traffic was present. Some significant interaction effects were also observed. The effect of absence of traffic was stronger when the sidewalk was even. When sidewalks were uneven, absence of traffic resulted in an increase of 0.30 points. However, when sidewalks were even, absence of traffic resulted in an increase of 0.71 points. The presence of benches was exclusively related to higher scores among participants who walked for transport more than 60 minutes/week. No relationships for the presence of benches were observed among participants who walked up to 60 minutes for transport. Streets in which the sidewalk was separated from traffic received on average 0.60 points more compared to streets without such separation.

**Table 2:** Results for the main and interaction effects of the environmental factors with individual and other environmental factors for sorting task B^1^.

	B	S.E.	p
Intercept	0.25	0.19	
**Main effects individual factors**			
Education (ref. = no or lower)			
−secondary	0.32	0.17	0.06 a
−tertiary	0.43	0.18	0.02 a
Current walking for transport^2^			
−≤60 minutes/week	−0.03	0.19	0.88 a
−>60 minutes/week	−0.31	0.17	0.07 a
**Main effects environmental factors** 3			
Sidewalk evenness	3.23	0.13	<0.001
Traffic	0.30	0.12	0.01
Benches	0.20	0.13	0.12
Separation from traffic	0.61	0.12	<0.001
**Interaction effects between environmental and individual factors**			
Benches*current walking for transport^1^			
Benches*≤60 minutes/week	0.05	0.22	0.82 a
Benches*>60 minutes/week	0.48	0.20	0.02 a
**Interaction effects between environmental factors**			
Sidewalk evenness*traffic	0.41	0.14	0.002

S.E. =  standard error.

1 In sorting task B one streetscape was manipulated on four environmental factors; sidewalk evenness, traffic, benches and separation from traffic. Sixty older adults sorted the streetscapes on their appeal for walking for transport using a seven-point scale. The B's can be interpreted as the effect of the environmental modification on this seven-point scale.

2 Reference category =  no walking for transport.

3 The reference categories for the environmental factors were the anticipated negative aspects of the factor (i.e. uneven sidewalk, no benches, no separation from traffic, and traffic present).

a The same superscripts indicate that the categories do not differ significantly.

### Results from qualitative analyses

Participants commented on sidewalk unevenness, traffic, upkeep, vegetation, separation from traffic, and benches. Often these comments reflected concerns for how these environmental characteristics became obstacles to walking.

#### Sidewalk evenness (sorting task A and B)

In both sorting tasks, sidewalk evenness was identified as one of the most critical environmental factors for almost all participants. Participants reported being afraid of falling and being injured when walking on uneven sidewalks. This is illustrated by the following quote: *“Those tiles are completely crooked, then you fall easily, that's dangerous to break a leg.” (sorting task A, man, 77 years)* Participants also discussed the importance of sidewalk evenness in light of their own or their peers' functional limitations: *“For me, the most important issue is accessibility, because I use a walking frame. I would like all of you to try out using a walker on a bad sidewalk. At that moment you don't care about traffic, because you are focusing upon where to place your feet and how to hold your walker.” (sorting task A, woman, 83 years)*

#### Traffic (sorting task A and B)

Overall, participants preferred streets without traffic over streets with traffic, because they liked to walk in calm streets or found it hazardous to cross busy streets. However, the presence of traffic was not the most influential factor. The following quote illustrates this: *“The main factor is the condition of the sidewalk. The second factor is the presence of obstacles (garbage) on the sidewalk. The third factor is the presence of traffic in the street. These are the three factors that guided me.”(sorting task A, man, 72 years)* Some participants indicated that the presence of traffic was less important because they regarded it as a temporary situation. Other participants regarded the presence of traffic as unavoidable: *“The presence of traffic also plays a role. For example, in this street there is no traffic whereas in that street there is, but you cannot avoid that. There are no streets anymore that are really free from traffic.” (sorting task B, man, 69 years)*

#### Overall upkeep (sorting task A)

Participants reported that the presence of garbage on the sidewalk was disliked for aesthetic reasons, but even more so for being an obstacle while walking on the sidewalk. The presence of broken windows and graffiti were also perceived as signs of antisocial behavior or irresponsible residents. Participants also mentioned poor upkeep of the street surface as dangerous for traffic accidents involving pedestrians or for falling when crossing the street. Despite the apparent importance of upkeep, it emerged as a less important factor than sidewalk evenness for most of the participants: *Interviewer: “What bothers you most: bad upkeep or an uneven sidewalk?” Participant: “In fact, an uneven sidewalk. You can clear up litter. However, they could repair a sidewalk as well, but that does not happen in reality.” (sorting task A, man, 69 years)*

#### Vegetation (sorting task A)

The presence of vegetation was generally considered to make a street more attractive and pleasant, however, this did not appear to be a main facilitator of walking for transport. This is illustrated by the following quote: *“I like the street with vegetation more, but vegetation is not a necessity to me.” (sorting task A, man, 72 years)* Some participants also mentioned trees being an obstacle on the sidewalk or their leaves causing slippery situations during autumn: *“During autumn, trees on the sidewalk might cause a fall hazard by making sidewalks slippery. On the other hand, trees might also be beautiful to walk along.” (sorting task A, woman, 65 years).*

#### Benches (sorting task B)

Benches were liked by participants because they provided the opportunity to sit down and rest. They enjoyed sitting on the bench while relaxing in the sun or socializing with neighbors. However, participants also mentioned that the benches could act as an obstacle on the sidewalk, for example: *“There are benches and a hedge which can hinder older adults while walking.” (sorting task B, male, 77 years)* Additionally, participants mentioned that the benches should be directed towards the street rather than to the houses.

#### Separation from traffic (sorting task B)

Generally, participants preferred streets in which sidewalks were separated from cyclists and motorized traffic by a hedge. The importance of being separated from cyclists rather than from cars was mentioned more often by participants. The hedges were considered important for safety reasons, and also because they added a natural element to the street. However, participants also mentioned that the hedge might act as an obstacle on the sidewalk. Furthermore, participants expressed a need for maintenance and continuity of the hedges: *“Such a hedge could be valuable but then it should be a continuous rather than an intermittent strip.” (sorting task B, male, 76 years)*

## Discussion

This study explored the use of photographs to investigate the effects of environmental manipulations on a street's appeal for walking for transport among older adults. Additionally, it studied the potential moderating effects of individual and other environmental factors on these relationships. Our quantitative and qualitative findings showed that sidewalk evenness was the most important environmental factor related to a street's appeal for walking for transport in older adults. This confirms findings from previous qualitative studies that highlighted the importance of sidewalk evenness for older adults' walking for transport [15]–[18]. Furthermore, our findings offer support to Alfonzo's [33] hypothesis that factors related to the need for comfort (such as sidewalk evenness) are more important for older adults' walking for transport than factors related to safety from crime and pleasurability. However, previous quantitative studies have not reported a consistent relationship between sidewalk evenness and older adults' walking for transport [19]. This might be explained by difficulties in assessing sidewalk evenness in real environments. Typically, perceived sidewalk quality is assessed by asking participants to rate the quality of the sidewalks in their neighborhood. However, sidewalk evenness might vary considerably from street to street within one neighborhood making it difficult for participants to provide an accurate answer to this question. In the current study, the target environment was made clear to participants by the presentation of photographs. It may be of value for future research to examine the perceived evenness of sidewalks in a participant's neighborhood (e.g., their residential street) using a survey recall measure compared with a photograph.

The effect of an even sidewalk on a street's appeal for walking for transport was even stronger when the street's overall upkeep was good and when traffic was absent. Upkeep and traffic might influence a street's appeal through several pathways, i.e. the need for comfort, crime- and traffic-related safety, and pleasurability. Our qualitative data indicated that the presence of garbage on the sidewalk was considered a potential obstacle. Hence, a ‘clean’ garbage-free street might further enhance the positive effect of an even sidewalk on a street's appeal. Likewise, the participants perceived streets without traffic as calm which might also make streets more comfortable to walk along.

Positive effects on appeal were also observed for separation from traffic and presence of benches. The presence of a hedge to separate the sidewalk from traffic gave the participants a feeling of protection from accidents with cars, but especially of protection from cyclists. The importance of potential interference with cyclists for older adults' transport walking has been reported previously in qualitative studies [15], [46], [47]. The presence of benches was only related to appeal among participants who walked for transport at least 60 minutes/week. Possibly, these participants might make longer walking trips which might increase the need to rest during a walk, and, hence, the need for a bench. While separation from traffic and benches were perceived as favorable, our qualitative data indicated that if these features were implemented, planners should ensure that they are not obstacles to walking on the sidewalk.

Based upon Alfonzo's hierarchy of walking needs, one would expect overall upkeep, vegetation, absence of traffic, and separation from traffic and benches to be unrelated to appeal for walking for transport when the sidewalk is uneven [33]. Our findings did not support this view; the positive effects of environmental factors on the appeal of the street for walking for transport was maintained across the levels of the other manipulated environmental factors. However, it was found that the effects of upkeep and traffic were stronger when sidewalks were even. This is positive news for intervention development as changes in the environmental factors all separately carry the potential to increase a street's appeal for walking for transport independent of other environmental characteristics present in that street. Nevertheless, our findings suggest that the most efficient way to increase a street's appeal for walking for transport is by providing even sidewalks.

There were no moderating effects by gender or functional limitations on the relationship between the micro-scale environment and the appeal of the street for walking for transport. This is promising for intervention development as it implies that the same environmental intervention would be equally effective for older men and women, and for those with and without functional limitations. However, the absence of other moderating effects in the current study might be explained by our small sample size and limited variance in the moderators and should be confirmed by future research in large samples.

The use of panoramic photographs enabled us to overcome several limitations of traditional research methods in this area. The use of photographs might be especially valuable to study micro-scale environmental characteristics, which appeared to be important for older adults' walking for transport in previous qualitative studies [15]–[18], but could not be confirmed by quantitative or real-world experimental research [19]. While it is possible to clearly represent most micro-scale environmental features in a photograph, some potentially important factors are more difficult to represent (i.e. cross-walks and traffic speed). The manipulation of a photographed street allowed us to control environmental (co)variation and to conduct a controlled experiment testing the effects of (combinations of) environmental factors on a street's appeal for walking for transport. To control for macro-environmental factors, our instructions for the sorting task included a high degree of standardization (e.g., ideal weather conditions and access- a walking duration of 10 minutes). This might have limited the generalizability of our findings. For example, rainy weather might increase the importance of sidewalk evenness [15] and micro-scale environmental characteristics might not relate to walking for transport if distance to destinations is too large [48].

Other limitations of the current study should also be acknowledged. The current study included a small convenience sample that was more active and highly educated than the general population of Flemish older adults [49], [50]; thus, this study should be considered exploratory. Further research in a larger, more representative sample is needed to confirm current findings. All photographs were manipulations of one street, which might limit the generalizability of our findings to other street configurations (e.g. single lane streets, streets without sidewalks). To minimize participant burden, the number of manipulated environmental factors was limited to four per sorting task. Although the environmental factors selected for manipulation appeared to be crucial in two previous studies [15], [40], there might be other factors that relate to a street's appeal for walking for transport (e.g. aesthetic features of houses). Furthermore, the effect of an environmental factor on the street score might depend on the magnitude of manipulation between the anticipated positive and negative aspects. Future research might expand the number of levels to study dose-response relationships between the environmental factors and appeal for walking for transport. The relationships of environmental factors with the appeal of micro-scale factors for walking for transport were studied, but these relationships were not extended to examining associations with actual walking for transport. Hence, future research is needed to confirm whether environmental factors related to the appeal of a streetscape are also related to actual walking for transport. Therefore, this type of mixed-methods approach using photographs should be regarded as a preliminary investigation that can inform research in real-life settings. It can inform which potential key environmental factors to focus on and how these might influence different PA behaviors.

To conclude, the protocols and methods used in the current study carry the potential to further our understanding of environment-PA relationships. Our findings indicated sidewalk evenness as the most important environmental factor influencing a street's appeal for walking for transport among older adults. However, future research in larger samples and in real-life settings is needed to confirm current findings.

## Supporting Information

Dataset S1 **Raw data obtained from the questionnaire and sorting tasks.**
(XLSX)
